# Clinical features of muscle cramp in 14 dogs

**DOI:** 10.1111/jvim.15965

**Published:** 2020-11-28

**Authors:** Teresa Gagliardo, Roberta Ruggeri, Andrea Di Paola, Massimo Baroni, Giunio B. Cherubini, Antonella Gallucci, Cristian Falzone, Stefania Trimboli, Andrey Albul, Gualtiero Gandini

**Affiliations:** ^1^ Department of Veterinary Medical Sciences University of Bologna Bologna Italy; ^2^ Clinica Veterinaria Valdinievole Monsummano Terme Pistoia Italy; ^3^ Department of Neurology and Neurosurgery Dick White Referrals Six Mile Bottom United Kingdom; ^4^ Centro Veterinario Professionale La Fenice Cagliari Italy; ^5^ Diagnostica Piccoli Animali Vicenza Italy; ^6^ Ambulatorio Veterinario Trimboli Messina Italy; ^7^ Veterinary Hospital of Neurology, Traumatology and Intensive Care Saint‐Petersburg Russia

**Keywords:** canine, hypocalcemia, hypoparathyroidism, muscle cramps

## Abstract

**Background:**

Muscle cramps (MCs) are prolonged, involuntary, painful muscle contractions characterized by an acute onset and short duration, caused by peripheral nerve hyperactivity.

**Objectives:**

To provide a detailed description of the clinical features and diagnostic findings in dogs affected by MCs.

**Animals:**

Fourteen dogs.

**Methods:**

Multicenter retrospective case series. Cases were recruited by a call to veterinary neurologists working in referral practices. Medical records and videotapes were searched for dogs showing MCs. The follow‐up was obtained by telephone communication with the owner and the referring veterinarian.

**Results:**

Three patterns of presentation were identified depending on the number of affected limbs and presence/absence of migration of MCs to other limbs. In 9/14 (64%) of dogs, MCs were triggered by prompting the dogs to move. 8/14 (58%) dogs were overtly painful with 6/14 (42%) showing mild discomfort. The cause of MCs was hypocalcemia in 11/14 (79%) dogs: 9 dogs were affected by primary hypoparathyrodism, 1 dog by intestinal lymphoma and 1 dog by protein losing enteropathy. In 3/14 cases (21%) the cause was not identified, and all 3 dogs were German Shepherds.

**Conclusions and Clinical Importance:**

Muscle cramps can manifest in 1 of 3 clinical patterns. Muscle cramps are elicited when dogs are encouraged to move and do not always appear as painful events, showing in some cases only discomfort. The main cause of MCs in this study was hypocalcemia consequent to primary hypoparathyroidism. In dogs having MCs of unknown etiology, idiopathic disease or paroxysmal dyskinesia could not be ruled out.

**Abbreviations**
EAMC: exercise associated muscle cramp
ELP: enteropathy losing protein
MC/MCs: muscle cramp/cramps
PMDs: paroxysmal movement disorders

## 1 INTRODUCTION

Muscle cramps (MCs) are prolonged, involuntary, painful muscle contractions, having acute onset and short duration, lasting from seconds to minutes.^1^ In human medicine, cramps are classified in “silent” and “true” cramps. Silent cramps have a normal electromyography, often develop after intense exercise, and are associated with metabolic myopathies where there is a defect in glycolysis or glycogenolysis.^2^ In dogs, a similar condition has not been described.

True MCs represent the expression of hyperexcitability of peripheral motor nerve.^3^ In human medicine, on electromyography, the true MCs are characterized by repetitive motor unit action potentials generated by simultaneous contraction of a large number of motor units. The number of motor units activated, and the frequency of their discharges increases gradually during the cramp (up to 150 Hz) and gradually stops according to the end of the event itself.^4^

As for humans, in veterinary medicine, MCs are considered the clinical manifestation of nerve hyperexcitability. However, electromyographic documentation of these events is missing.^3^

In humans, the diseases most commonly associated with MCs include disorders of the lower motor neuron (eg, amyotrophic lateral sclerosis, radiculopathies, neuropathies), metabolic disorders (eg, pregnancy, uremia, hypothyroidism, hypoadrenocorticism, hyperparathyroidism), conditions leading to the acute depletion of extracellular volume (eg, vomiting, diarrhea, diuretic therapies, hemodialysis), the use of certain drugs (eg, nifedipine, salbutamol, terbutaline, alcohol), and idiopathic forms (eg, exercise‐related cramps and nocturnal cramps in elderly subjects).^5^

Several disorders can result in episodic paroxysmal events that might mimic MCs, including seizures (epileptic and nonepileptic) or involuntary paroxysmal movement disorders (PMDs).^6^ Due to the lack of reliable methods for the differentiation between epileptic and nonepileptic paroxysms, proper assessment of these episodes is sometimes challenging.^7^

To date, in veterinary medicine, there is a lack of detailed description of MCs, limited to a few dogs affected by hypocalcemia and a single report of 2 poodles with hypoadrenocorticism.^8^, ^9^ The aim of this retrospective study was to provide a detailed description of the clinical features of MCs, assess the clinicopathological and diagnostic abnormalities, and describe the treatment and clinical course of the affected dogs.

## 2 MATERIALS AND METHODS

Medical records of dogs with muscle cramps, presented at the Veterinary Teaching Hospital of the University of Bologna from January 2010 and May 2019, were retrospectively reviewed. Additionally, cases were recruited by a call to veterinary neurologists working in selected referral practices.

Dogs were included if they had video footage (10/14) or analysis of medical records and detailed phone questioning of the owner and veterinarian (4/14).

To be enrolled in the study, the medical record had to include: information about signalment (breed, age, sex, weight); age at the first MC episode; MCs frequency; duration of a MC episode; description of possible triggers or conditions associated with the occurrence of MCs; time from the first MCs episode to the diagnosis. Due to the multicentric origin of the study, a minimum data base blood tests comprising hematology, biochemical analysis (including AST, ALT, SAP, GGT, creatinine, urea, bilirubin, protein, albumin, CK) and blood gas analysis was required. To be included in the study, serum ionized calcium concentration results had to be available for each dog.

Dogs with paroxysmal events associated with urination, defecation, hypersalivation, or loss of consciousness were excluded from the study. Additionally, dogs with cardiovascular, respiratory, and orthopedic conditions potentially mimicking MCs were excluded based on medical records, clinical examination, and diagnostic findings.

Information concerning the presence of any other sign, concurrent medical conditions or the administration of drugs was collected. Any other diagnostic investigation performed (urine analysis, parathormone concentration, abdominal ultrasound, electrodiagnostics test) was evaluated.

The follow‐up, including information about the treatment, was obtained by telephone communication with the owner or the referring veterinarian.

The data collected were inserted into an Excel file (Microsoft) and analyzed using a commercial statistical data analysis software (Prism 7.0a, GraphPad Software, Inc, San Diego, California).

The Shapiro‐Wilk test was used to assess the normality of continuous data. Mean and SD calculations were reported for normally distributed data. Otherwise, the median and range were reported.

The *t* test was used to compare the serum ionized calcium concentration among the different patterns of clinical presentation and continuous variable between the group of hypocalcemic dogs and the group of dogs with MCs of unknown origin. According to the disease underlying MCs, dogs were divided into 2 groups: dogs with primary hypoparathyroidism vs other causes. The continuous variables (ionized calcium, total calcium, phosphorus, and magnesium) were compared using the Mann‐Whitney test while the categorical variables (number of affected limbs and pain manifestations) were compared using the Fisher's exact test. The *P*‐value <.05 was considered statistically significant.

## 3 RESULTS

### 3.1 Animals

Fourteen dogs were included in the study (Table 1). Breeds represented included: 5 German Shepherd (36%), 3 mix‐breed (22%), 1 English bulldog (7%), 1 Labrador (7%), 1 Lurcher (7%), 1 Whippet (7%), 1 Border collie (7%), 1 Pinscher (7%). Four were females (1 spayed) and 10 males (3 neutered). The mean weight at the first neurological evaluation was 26.1 kg (±11.23). The mean age at first MC was 90.2 months (±42.3).

**TABLE 1:** Comparison of signalment, clinical features, and diagnostic findings between hypocalcemic and nonhypocalcemic dogs

	Hypocalcemic dogs (n = 11)	Nonhypocalcemic dog (n = 3)
Breed	3 Mixed breeds 1 English bulldog 2 German Shepherd 1 Border collie 1 Labrador 1 Pinscher 1 Whippet 1 Lurcher	3 German Shepherd
Sex	Males: 7 (neutered: 2) Females: 4 (spayed:1)	Males: 3 (neutered: 1) Females: 0
Weight	23.2 (±10.7)	37 (±4.3)
Age at first muscle cramp	78.8 (±38.2) months	132 (±30.8) months
Trigger	Stress/excitement: 3 dogs Exercise: 6 dogs Unknown information: 2 dogs	Exercise: 2 dogs At rest: 1 dog
Type of PATTERN	I: 5 II:4 III: 2	I: 0 II: 0 III: 3
Presence of pain	Overt pain: 5 dogs Discomfort: 6 dogs	Overt pain: 3 dogs
Median duration of signs before the diagnosis	30 days (range, 1‐365)	6 days (range, 3‐7)
Total calcium value (mean ± SD)	5.6 mg/dL (±1.7)	10 mg/dL (±0.4)
Phosphorus value (mean ± SD)	5.9 mg/dL (±1.9) in 10 dogs	4.25 mg/dL (±0.9) in 2 dogs
Magnesium value (mean ± SD)	1.8 mg/dL (±0.5) in 5 dogs	1.8 mg/dL (±0.1) in 2 dogs
Diagnosis	9 primary hypoparathyrodism 1 PLE 1 intestinal lymphoma	3 unknown origin

### 3.2 History and clinical features

The MCs frequency varied from 1 every 3 months to 10 episodes per day. In the majority of the cases (12 dogs), MCs were multiple in a day. Only in 2 dogs, MCs occurred occasionally. The duration of MCs events varied from a few seconds to 15‐20 minutes. The median time of the occurrence of the first MCs preceding the diagnosis was 67 days (range, 0‐365).

On owners' observation, all dogs were alert and responsive during and between episodes. Eight dogs (57%) showed MCs with moderate/intense exercise (while playing or running), 3 (21%) when simply stressed or restless. For the remaining 3 dogs (21%) this information was unknown. In 9 dogs (64%), MCs trigger consisted in prompting the dogs to move.

In 13 dogs (93%) the general physical examination was unremarkable. One dog had an abnormal Body Condition Score (BCS = 2/9). The parts of the neurological examination performed at rest were normal in all 14 dogs, except for the recognition of MCs, which obviously affected the gait. When prompted to move, gait evaluation was abnormal in 11 dogs (78%), especially at quicker paces, showing cramping episodes within a few minutes of exercise. Three dogs (22%) were not moved because they were already showing MCs. In all cases, MCs resolved after a few minutes of rest.

In 5 cases, the progression of the physical activity induced a migration of the MCs from 1 limb to another. In 3 dogs, fasciculations shortly preceded the MCs. In 1 dog, due to the prolongation of exercise prompting, MCs evolved in a dramatic tetanic seizure (Supporting Information Video S1).

Three different clinical patterns of MCs presentation were identified: PATTERN I (migrating pattern), present in 5 dogs, was characterized by a sudden onset of stiffness and abduction of 1 thoracic limb, failure to bear the weight lasting few seconds, followed by a sustained muscular contraction with flexion of 1 pelvic limb and fall in lateral recumbency (Supporting Information Video S2). PATTERN II (pelvic limbs pattern) consisted of a prolonged muscular contraction and flexion of the pelvic limbs. When these dogs were forced to continue physical activity, MCs migrated between the pelvic limbs, without involvement of thoracic limbs (Supporting Information Video S3). This pattern was present in 4 dogs.

Five dogs showed the PATTERN III (single‐limb pattern). In these cases, MCs involved only 1 limb (thoracic limb: 3 dogs; pelvic limb: 2 dogs) and were characterized by an abrupt contraction and flexion of the single limb, without any migration toward other limbs (Supporting Information Video S4).

Eight dogs (58%) showed an overt pain characterized by increasing yelps, tachypnoea, and restlessness during the episodes. Six dogs (42%) exhibited only signs of discomfort, consisting in marked tachypnoea, restlessness, swallowing, and lips licking.

Concerning the cases with longer history of MCs activity (5/14 dogs), between MCs onset and the diagnosis, 1 dog was treated by the referring vet with meloxicam (0.1 mg/kg q24h OS) and tramadol (2.5 mg/kg q12h OS), suspecting paroxysmal pain events due to the rupture of the cranial cruciate ligament. Two dogs received phenobarbital (3 mg/kg q12h OS) associated in 1 case with amitriptyline (1 mg/kg q12h OS) and prednisolone (1 mg/kg q24h OS), in the suspicion of seizures. One further dog was treated with prednisolone (1 mg/kg q24h OS) and oclacitinib (0.4 mg/kg q12h OS) due to concomitant dermatitis. None of them received drugs potentially associated with the development of MCs.

### 3.3 Investigation and diagnosis

After the completion of the diagnostic work‐up, 11 dogs were hypocalcemic and for 3 dogs the etiological diagnosis remained unknown. Among the hypocalcemic dogs, hypocalcemia was due to primary hypoparathyroidism in 9 cases. The other 2 dogs underwent an intestinal biopsy resulting affected by an intestinal lymphoma and a protein‐losing enteropathy (PLE), respectively. These 2 latter cases had a marked decrease of serum albumin and total protein concentrations.

In hypocalcemic dogs, the mean value of total calcium (measured in 12 dogs) and ionized calcium was 5.6 mg/dL (±1.7; normal reference interval: 8.5‐10.5) and 0.7 mmoL/L (±0.2; normal reference interval: 1.2‐1.4). The mean value of ionized calcium in PATTERN I (migrating pattern) dogs was 0.6 mmoL/L (±0.1); in PATTERN II (pelvic limbs pattern) dogs was 0.8 mmoL/L (±0.2) and in PATTERN III (single‐limb pattern) dogs was 1.0 mmoL/L (±0.2). A significant difference (*P* = .02) was found for ionized calcium concentration between dogs exhibiting PATTERNS I and III.

Serum parathormone concentration was available in 9 dogs. It was measured using radioimmunoassay, ELISA, and chemiluminescent assay respectively in 5, 3, and 1 dog. The median serum parathormone concentration value was 15.25 pg/mL (range, 0.9‐64; reference value: 20‐65). All these dogs resulted affected by primary hypoparathyroidism, including a dog presenting serum parathormone concentration within normal limits (64 pg/mL). In this latter case, parathormone concentration was judged as inappropriate considering the extremely low level of serum ionized calcium (0.6 mmoL/L) exhibited by the dog.

The biochemical profile showed an increase in ALT and AST in 10 and 5 dogs, respectively. In all 14 dogs CK was measured, the median value was 172 IU/L (range, 68‐1035; reference value: 50‐290). In 12 dogs, the biochemical analysis comprised the measurement of phosphorus (mean 5.6 mg/dL; ±1.8) and in 7 cases of magnesium (mean 1.8 mg/dL; ±0.4).

Abdominal US was performed in 7 cases; 2 dogs presented an abnormality of the intestinal wall; 5 dogs did not show any alteration. Urinalysis was performed in 5 dogs, resulting normal in 1 case. Two dogs had a decreased urinary specificity gravity, 1 dog had an increased urinary specificity gravity, 1 dog had a urinary infection due to *Pseudomonas aeruginosa*.

The 3 dogs with unknown etiological diagnosis were German Shepherds (Table 1). All of them were male and were significantly older at MCs onset than hypocalcemic dogs (*P* = .03). The duration of clinical signs before the diagnosis was not found statistically different between the 2 groups (*P* = .2).

All these 3 dogs showed PATTERN III (single limb pattern) MCs. One dog had MCs in the pelvic limbs (both left and right) and 2 dogs had MCs in the thoracic limb (1 in the right limb and the other in both limbs). All these dogs had a quite short period of occurrence of MCs (mean time from cramps onset to remission 7.6 days; ±2). For this main reason, no electrodiagnostic investigation and/or advanced diagnostic imaging was performed to exclude possible focal lower motor neuron involvement. Two dogs exhibited MCs after a vigorous exercise, 1 dog at rest. In a dog, MCs relapsed after 1 year and 6 months from the first evaluation. The owner reported that MCs were less severe than the first episodes, lasted for a few days, and eventually disappeared spontaneously. The 2 other dogs did not show further cramps. One dog died 6 months later after being hit by a car and the other dog died 3 years later from unrelated causes.

Considering the group of dogs affected by primary hypoparathyroidism vs other causes, the total calcium (*P* = .04) and ionized calcium (*P* = .02) were significantly different between the 2 groups, being significantly lower in the group of the hypoparathyroid dogs. No significant differences were found for pain (*P* = .07), albumin, magnesium, and phosphorus (*P* = .9) between the 2 groups.

### 3.4 Treatment and outcome

Dogs with MCs of unknown origin received no treatment and the owners referred spontaneous disappearance of MCs in all the cases.

In the group of the 11 hypocalcaemic dogs, 4 received an emergency treatment based on the IV administration of 10% calcium gluconate. Afterward, they were treated with calcitriol PO (0.01‐0.02 mcg/kg q24h) and 2 of them also with oral supplementation of calcium carbonate (1000‐4000 mg q24h). Seven dogs did not need emergency treatment, and therapy consisted of the administration of oral calcitriol (4 dogs) and dihydrotachysterol (3 dogs). In all cases, a resolution of the clinical signs occurred after normalization of serum calcium levels. All the treated dogs showed no further relapses.

## 4 DISCUSSION

This study produced an accurate description of the clinical findings of dogs suffering from MCs. The retrospective evaluation of the video recording and the detailed description of the events permitted to identify 3 clinical patterns of presentation depending on the presence (PATTERNS I and II/migrating and pelvic limbs pattern) or absence (PATTERN III/single‐limb pattern) of MCs migration to other limbs. The migration of the cramp usually occurred when dogs were forced to continue the physical activity. During the examination, for ethical reasons and to not cause suffering to the animal, the movement was not always carried out beyond the necessity to show the presence of MCs. Therefore, it was not always possible to determine whether a pattern remained stable or, increasing the exercise, evolved into another pattern.

In hypocalcemic dogs, ionized calcium concentration was significantly different between PATTERNS I and III, suggesting that lower ionized calcium levels could produce more severe signs.

In the majority of the cases, MCs were triggered by the exercise, such as running or playing. Unfortunately, due to the retrospective nature of the study, we have not objectively assessed the presence of anxiety and other possible triggers. However, restlessness and hyperexcitation occur in cases of hypocalcemia.^10^

Interestingly, MCs were not always accompanied by obvious signs of pain. According to the definition adapted from humans, the key feature of the MC should be the presence of pain.^1^ However, dogs can minimize the signs of their discomforting condition. Licking of the lips, yawning, tachypnoea, and concerned expression can be the only clinical indicators of a dog suffering from persistent discomfort.

The recognition of MCs can be challenging. The suspicion of the occurrence of MCs is mainly based on the observation and knowledge of the clinical presentation. Clinicians unconfident with this unusual disorder might misdiagnose MCs as orthopedic disease, epileptic seizures or other PMDs.

In 1 hypocalcemic dog of our series, MCs were initially misdiagnosed as intermittent lameness due to patellar luxation and the dog was unsuccessfully treated with NSAID for several months.

In dogs with episodic lameness of undefined origin, there should always be assessment of the serum ionized calcium in the diagnostic work‐up.

The PATTERN I (migrating pattern), characterized by a sustained muscular contraction migrating among the 4 limbs followed by falling in lateral recumbency with vocalization, might be misdiagnosed as an epileptic seizure. The preservation of the consciousness and the lack of autonomic signs should orient toward the exclusion of an epileptic seizure.

Muscle cramps occurring as for the PATTERN III (single‐limb pattern) could be even more difficult to be differentiated from a paroxysmal movement disorder (PMD). In veterinary medicine, paroxysmal dyskinesia has been used as a broad term to describe an abnormal, sudden, involuntary contraction of a group of skeletal muscles recurring episodically.^11^ Similar to MCs, PMDs are not characterized by autonomic signs, consciousness is not impaired and abnormal postictal behavior is not observed.^12^ Unlike MCs, PMDs are considered painless.^12^ Nevertheless, even if not showing overt pain, dogs affected by PMDs could show signs of discomfort.^13^

The objective demonstration of MCs in veterinary medicine remains difficult. Despite they present specific features on electromyography, this diagnostic test in dogs requires anesthesia and it is almost impossible to be performed during the attacks.^3^

In our study, the etiological diagnosis of the MCs was not obtained in 3 dogs. All of them were male German Shepherds and had a PATTERN III (single‐limb pattern) clinical presentation. They were suspected to suffer from MCs because the cramping attacks were almost identical to those of hypocalcemic dogs showing overt pain during the episode. These dogs did not undergo a complete diagnostic work‐up including MRI or electrodiagnostic tests, therefore other causes, as PMDs, seizures, or MCs secondary to lower motor neuron diseases, could not be entirely excluded.

Despite not previously reported in veterinary medicine, it is not to reject the hypothesis that MCs, in this group of dogs, had an idiopathic origin. In people, when no causes are identified MCs are classified as idiopathic.^14^

Idiopathic cramps notably include nocturnal leg cramps.^2^, ^15^ Although nocturnal muscle cramps occur at night, 20% of cases can have leg cramps primarily during the daytime.^16^ Nocturnal leg cramps are more common in the elderly and typically involve the calf or foot muscles, frequently awaken the patient from sleep. The current hypothesis includes that they are secondary to the loss of motor neurons innervating the affected muscles and represent a similar phenomenon as that observed in patients with amyotrophic lateral sclerosis.^5^

Various pharmacologic treatments have been studied for nocturnal leg cramps, including quinine. However, due to the severe adverse effect of quinine, stretching exercises are the sole indication to reduce MCs.^5^ It is interesting to notice that all the dogs with MCs of unknown origin herein described, were significantly older than hypocalcemic dogs.

Two out of 3 dogs with MCs of unknown origin exhibited MCs during the exercise. In human medicine, exercise associated MCs (EAMC) are very common and their etiology is not fully understood. The proposed theories include dehydration, altered plasma electrolyte concentrations, or α‐motor neuron hyperexcitability. In this type of MCs, stretching relieves the signs without altering hydration or electrolyte status^,^ demonstrating that restoring electrolyte and fluid balance is not a requisite for alleviating EAMC. Therefore, a neural mechanism, linked to α‐motor neuron hyperexcitability, has been proposed.^17^

The most common cause of MCs in this study was hypocalcemia. According to the literature, dogs with chronic hypocalcemia display intermittent clinical signs, usually following periods of exercise or excitement.^18^

The most common causes of hypocalcemia in dogs are hypoalbuminemia and renal failure.^19^ Interestingly, in our case series, these conditions were uncommon, and 9 out of 11 hypocalcemic dogs were affected by primary hypoparathyroidism, a much rarer disease, possibly producing lower concentrations of ionized Calcium.^20^

Hypomagnesemia can result in hypocalcemia.^19^ Magnesium is an important cofactor for PTH secretion. In conditions of severe magnesium deficiency, parathyroid secretion is suppressed inducing hypocalcemia and hyperphosphatemia. Hypomagnesemia also induces a reversible resistance to the actions of PTH at the level of both bone and kidney.^21^ In human medicine, hypomagnesemia is frequently undetected. Many patients with magnesium deficiency remain asymptomatic until serum magnesium concentration decrease to 0.5 mmoL/L or lower.^22^ The earliest manifestations of magnesium deficiency are neuromuscular and neuropsychiatric signs. The most common clinical manifestations result from hyper‐excitability, including positive Chvostek's and Trousseau's signs, tremor, fasciculation, and tetany.^23^

In this case series, magnesium was measured in 7 dogs, and it was found decreased only in the hypocalcemic dog affected by PLE. Unfortunately, our data are too limited to evaluate the possible role of magnesium in the onset of muscle cramps.

The major limitation of the present study consists of the small number of the dogs included in the study, which prevents to answer to many of the remaining open questions on MCs. For the same reason, statistical analysis was very limited and did not include breed and sex. Nevertheless, for the descriptive purposes of the study, it could be considered adequate, and robust statistical analysis not essential. It should be emphasized that it is extremely difficult to collect a relevant number of cases of MCs due to the rarity of this clinical manifestation. An additional limitation can be found in the variability of the diagnostic and therapeutic protocols due to the retrospective multicentric nature of the study.

## CONFLICT OF INTEREST DECLARATION

Authors declare no conflict of interest.

## OFF‐LABEL ANTIMICROBIAL DECLARATION

Authors declare no off‐label use of antimicrobials.

## INSTITUTIONAL ANIMAL CARE AND USE COMMITTEE (IACUC) OR OTHER APPROVAL DECLARATION

Authors declare no IACUC or other approval was needed.

## HUMAN ETHICS APPROVAL DECLARATION

Authors declare human ethics approval was not needed for this study.

## Supporting information


**Video S1** Dramatic tetanic seizure in a hypocalcemic dog.

**Video S2** Dog showed a sudden onset characterized by initial stiffness and abduction of 1 front limb, failure to bear the weight lasting few second, fall in lateral recumbency and sustained muscular contraction with flexion of 1 hind limb (PATTERN I).

**Video S3** Dog showed a prolonged muscular contraction and flexion of the hindlimbs. When this dog was forced to continue physical activity, MCs migrated between the pelvic limbs, without involvement of thoracic limbs (PATTERN II).

**Video S4** MC involved only 1 limb, characterized by an abrupt contraction of the single limb, without any migration toward other limbs (PATTERN III).
